# Psychiatric Manifestations of Coeliac Disease, a Systematic Review and Meta-Analysis

**DOI:** 10.3390/nu12010142

**Published:** 2020-01-04

**Authors:** Emma Clappison, Marios Hadjivassiliou, Panagiotis Zis

**Affiliations:** 1 Medical School, University of Sheffield, Sheffield S10 2YN, UK; 2 Academic Department of Neurosciences, Sheffield Teaching Hospitals NHS Foundation Trust and University of Sheffield, Sheffield S10 2JF, UK; m.hadjivassiliou@sheffield.ac.uk

**Keywords:** coeliac disease, gluten free diet, psychiatric manifestations, autistic spectrum disorder, attention deficit hyperactivity disorder, depression, anxiety, bipolar disorder, schizophrenia, eating disorders

## Abstract

Background: Coeliac disease (CD) is increasingly prevalent and is associated with both gastrointestinal (GI) and extra-intestinal manifestations. Psychiatric disorders are amongst extra-intestinal manifestations proposed. The relationship between CD and such psychiatric disorders is not well recognised or understood. Aim: The aim of this systematic review and meta-analysis was to provide a greater understanding of the existing evidence and theories surrounding psychiatric manifestations of CD. Methodology: An online literature search using PubMed was conducted, the prevalence data for both CD and psychiatric disorders was extracted from eligible articles. Meta analyses on odds ratios were also performed. Results: A total of 37 articles were included in this review. A significant increase in risk was detected for autistic spectrum disorder (OR 1.53, 95% CI 1.24–1.88, *p* < 0.0001), attention deficit hyperactivity disorder (OR 1.39, 95% CI 1.18–1.63, *p* < 0.0001), depression (OR 2.17, 95% CI 2.17–11.15, *p* < 0.0001), anxiety (OR 6.03, 95% CI 2.22–16.35, *p* < 0.0001), and eating disorders (OR 1.62, 95% CI 1.37–1.91, *p* < 0.00001) amongst the CD population compared to healthy controls. No significant differences were found for bipolar disorder (OR 2.35, 95% CI 2.29–19.21, *p* = 0.43) or schizophrenia (OR 0.46, 95% CI 0.02–10.18, *p* = 0.62). Conclusion: CD is associated with an increased risk of depression, anxiety, eating disorders as well as ASD and ADHD. More research is required to investigate specific biological explanations as well as any effect of gluten free diet.

## 1. Introduction

The prevalence of CD is 1% in the Western population and it is increasing amongst both pediatric and adult populations [1,2,3]. Possible explanations for this increase include easier diagnostic methods and better targeted screening [4,5]. In addition to classic gastrointestinal (GI) symptoms, extra-intestinal symptoms such as neurological, psychiatric, and skin related are increasingly recognised [1,6,7,8,9]. These extra-intestinal symptoms when presenting in isolation are challenging in the diagnosis of CD [1,10].

Psychiatric disorders often reported in the literature include autistic spectrum disorder (ASD), attention deficit hyperactivity disorder (ADHD), bipolar disorder, depression, anxiety, schizophrenia, other psychotic disorders and eating disorders [1,7,8,10,11,12,13,14,15]. These psychiatric disorders are therefore the focus of this systematic review and meta-analysis.

### Interaction between CD and Psychiatric Disorders

A complex interaction between CD and such psychiatric disorders is proposed in the literature [10,12,15,16]. Theories are often split into specific and non-specific mechanisms [16]. Specific mechanisms refer to biological processes that may be producing overlapping pathologies, such as speculation over a direct ‘gut–brain’ relationship [12,16,17]. Non-specific mechanisms include the social and emotional consequences of CD diagnosis [7].

A strict gluten-free diet (GFD) is the only effective treatment for CD and this is often claimed to influence the risk of psychiatric disorders, but the exact role of the GFD has not been investigated in detail [7,8,10]. Some propose that the improvement of the GI symptoms with GFD may be protective against the development of psychiatric disorders [1]. However, there are also claims that it may increase such risk due to the detrimental effect of GFD on quality of life [1,7]. Conversely, psychiatric disorders can hinder adherence to the GFD, suggesting a need for the appropriate treatment of psychiatric issues in order to improve overall outcomes [7].

The proposal of a direct gut–brain relationship contributing to the pathophysiology, commonly features in literature, in particular in reference to schizophrenia and ASD [1,12,16,18,19,20,21]. Theories often describe autoimmunity and inflammation as potentially playing a role [11,22,23]. Other theories highlight the fact that the gastrointestinal tract is the region of entry of many substances that may be implicated to psychiatric pathology [19]. Furthermore, the ingestion and breakdown of gluten into immunogenic peptides leaking through the intestinal wall and getting into the brain may potentially interfere with its functioning [12,13,19,20,21,24].

Endogenous essential amino acids, such as tryptophan are known to be crucial in the production of serotonin. Despite being located in the gut, serotonin also plays an important role in mood regulation and cognition, whilst enabling GI regulation [25]. For example, Groer et al., (2018) proposed that insufficient tryptophan levels are associated with obesity and inflammation and increase risk of maternal depression in obese pregnant women [26].

Dehhaghi, Kazemi Shariat Panahi, and Guillemin (2019) discuss evidence concerning molecular communications between microbiota within the gut and the CNS, explaining how poor integrity of the intestinal barrier contributes to poor CNS function, which has a subsequent influence on mood and behaviour.

Healthy gut microbiota is vital in the protection against both psychiatric disorders, as well as GI disorders such as CD [25]. Altered gut microbiota have been identified in individuals with CD, indicating this as a partial cause of inflammatory responses to gluten [27]. Sacchetti and Nardelli (2019) argue a relationship between gut microbiota and CD, however they also acknowledge that CD is a multifactorial disease and therefore this alone does not fully explain such manifestations. Evidence suggests that gut microbiota has the ability to also influence mood and behaviour, as it has been implicated in psychiatric disorders, such as anxiety and depression [25]. Individuals with depression have been found to possess different gut microbiota to those without [25]. There is therefore evidence of an important interaction between the brain and the gut that could potentially add to the pathophysiology of such extraintestinal manifestations.

Despite a long history of research investigating associations between CD and psychiatric disorders the literature is often conflicting, regularly concluding that there is limited knowledge and highlighting the need for further investigations [1,16,17,19,22,28,29,30]. Additionally, small sample studies limit the reliability and generalisability of these findings [28].

The aim of this systematic review and meta-analysis is to overview the existing literature on coeliac diseases and psychiatric disorders. Furthermore, we wanted to determine the prevalence each psychiatric disorder in patients with coeliac disease and vice versa in order to calculate the respective odds ratios to have on disorder when suffering from the other.

## 2. Materials and Methods

### 2.1. Literature Search Strategy

A systematic computer-based literature search using the PubMed database was conducted on the 14 May 2019. For the search, we used two Medical Subject Headings (MeSH) terms. Term A was “Celiac” or “Coeliac”. Term B was “Psychiatric” or “Depression” or “Depressive” or “Psychosis” or “Psychotic” or “Schizophrenia” or “Schizoaffective” or “Anxiety” or “Mood disorder” or “Mood disorders” or “Autism” or “Autistic” or “Asperger” or “Asperger’s” or “Anorexia” or “Anorexic” or “Bulimia” or “Bulimic” or “Eating disorder” or “Eating disorders” or “Bipolar” or “Manic” or “Mania” or “Hypomanic” or “Hypomania” or “ADHD” or “Attention Deficit Hyperactivity disorder” or “PTSD” or “Stress disorder”. Three filters were applied; English language, human participants, and full text availability. We also perused the reference lists of the papers so as to try and include further relevant paper that were not identified with the above-mentioned search strategy.

### 2.2. Inclusion and Exclusion Criteria

1. Articles needed to provide original data.
2. Articles needed to concern the relationship of CD and psychiatric disorders.
3. CD should have been confirmed, either serologically with anti-endomysial (EMA), or a duodenum biopsy.
4. Formal diagnosis of psychiatric disorders should have been made.

All articles were abstract screened by a minimum of three authors in a blinded fashion using Rayyan software to ensure accuracy. Those found to meet any of the exclusion criteria were removed and any conflicts were settled by consensus during a face-to-face meeting in which the abstracts were reread. All remaining papers were screened again as a full article by at least two authors and conflicts were settled as before. Where a paper was not available online, a university interlibrary request was made for the item, a British Library request and failing these we attempted to find the authors contact details.

**Figure 1:**
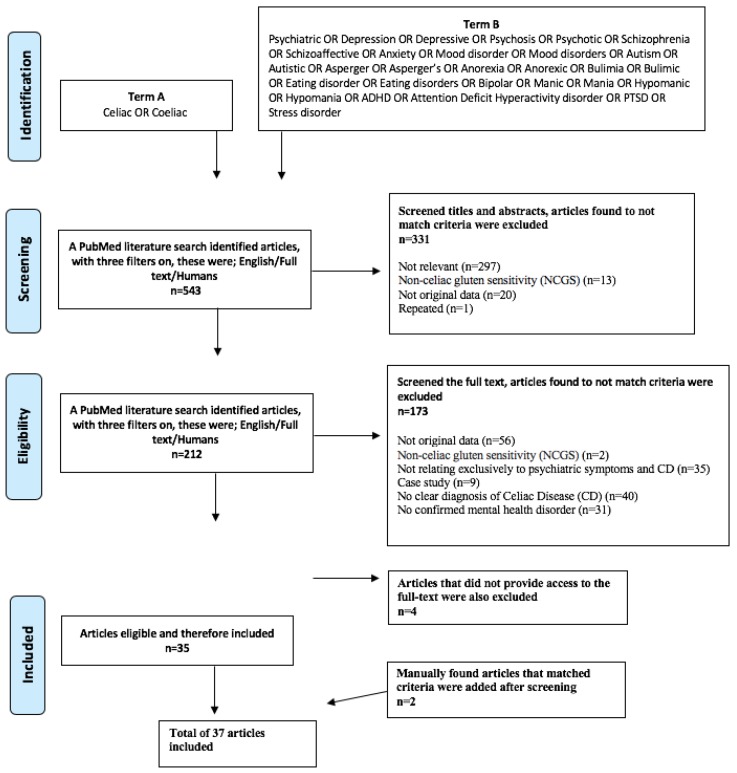
PRISMA flow chart displaying this selection process.

Figure 1 contains a Preferred Reporting Items for Systematic Reviews and Meta-Analysis (PRISMA) flow chart displaying this process.

### 2.3. Statistical Analyses

A database was developed using IBM SPSS Statistics (version 23.0 for Mac). Data was extracted from each study and included: study type, population size, type of psychiatric disorder, prevalence of the psychiatric disorder, whether this concerned an adult or pediatric population, and information about GFD. Frequencies and descriptive statistics were examined for each variable. The outcomes of interest were the proportion of patients with CD suffering from each psychiatric disorder and the proportion of patients suffering from each psychiatric disorder that had CD.

The meta-analysis of odds ratios was conducted using the RevMan program (RevMan, 2014) as suggested by the Cochrane Collaboration Group. Heterogeneity between studies was assessed using the I2 statistic. Data were analysed using a random effects model.

A value of *p* < 0.05 was considered to be statistically significant.

### 2.4. Compliance with Ethical Guidelines

This article is based upon previously published studies. The article follows the journal’s ethical guidelines.

## 3. Results

A total of 543 articles were identified following this search, 331 were excluded due to not matching criteria based on titles and abstracts alone. A second screening of the full-text on the remaining 212 resulted in 173 articles being excluded. A further four was excluded due to not providing the full text, leaving 35 articles eligible to be included in this review. Another two articles were manually found during this screening process that also fitted the criteria. Therefore, a total of 37 articles that matched inclusion criteria were identified to be included in this review (Figure 1). Table 1 represents a summary of the descriptive characteristics of these studies included in this review.

The 37 articles were categorised according to specific psychiatric disorders and these included autistic spectrum disorders (ASD), attention deficit hyperactivity disorder (ADHD), schizophrenia or other psychotic disorders, depression, anxiety, bipolar disorder, and eating disorders. Articles were then analysed according to the prevalence of these psychiatric disorders in patients with CD and vice versa. However, no articles investigated CD amongst patients with depression, anxiety, bipolar disorder, or schizophrenia, therefore the pooled prevalence of CD within these disorders was not calculated. Additionally, a total of 15 articles also investigated the role of the GFD in such disorders and these findings were also examined.

### 3.1. ASD and ADHD

ASD literature consisted of nine articles in total and comprised of 39,207 participants [31,32,33,34,35,36,37,38,39]. Only one found statistically significant findings, therefore only one concluded the need for routine CD screening within the ASD population [31].

**Figure 2:**
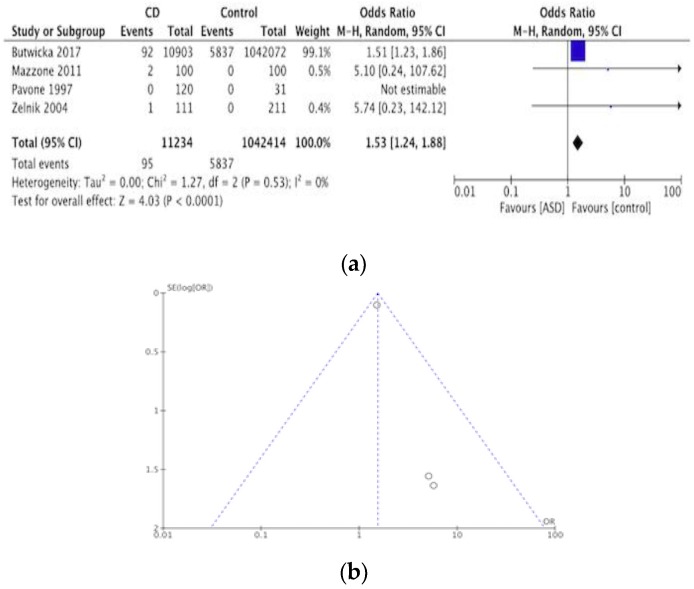
(**a**) Forest plot of pooled prevalence of ASD in CD. (**b**) Funnel plot investigating distribution in ASD studies.

This included 38,440 participants with a diagnosis of CD. In total, 3336 were found to have ASD making the pooled prevalence of ASD in CD 8.7%. Such information about the prevalence of ASD in CD was available through two cross-sectional [32,33] and four case-controlled studies [34,35,36,37]. The meta-analysis of the four case-controlled studies is summarized in a forest plot in Figure 2a, the odds of having ASD was significantly higher in the CD groups compared to controls (OR 1.53, 95% CI 1.24–1.88, *p* < 0.0001). Figure 2b shows a funnel plot in which presents heterogeneity in the studies included.

Investigation into the prevalence of CD in patients with ASD was done by three cross sectional studies [33,38,39] and two case-controlled studies [31,37]. Of the 767 ASD participants, ten were found to have CD making the pooled prevalence of CD in ASD 1.3%. All of these individuals came from the same study, which is the only one that confirmed significant findings [31]. In addition to this, Juncia et al. (2018) noted GI symptoms in 34% of a pediatric ASD sample. Józefczuk et al. (2018) found no difference between the presence of CD-specific antibodies in ASD patients and controls, or any deficits in intestinal permeability.

**Figure 3:**
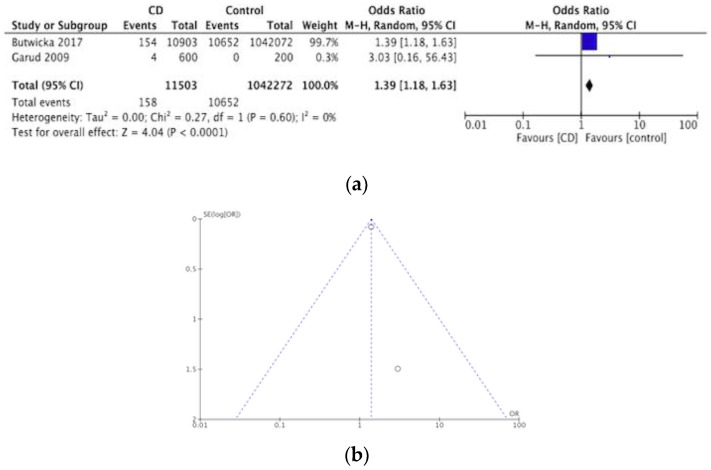
(**a**) Forest plot of pooled prevalence of ADHD in CD. (**b**) Funnel plot investigating distribution in ADHD studies.

Out of eight articles on ADHD, two concluded a significant association between ADHD and CD [34,40]. One of these referred to a sample size of eight participants of which two (siblings) were found to have ADHD as an initial presentation of CD [40]. The eight articles included a total of 12,366 participants. The prevalence of ADHD in CD was assessed by one case series study [41] two cross sectional studies [42,43] two case-controlled studies [34,44] and one cohort study [40]. Out of 11,965 CD participants, 165 were found to have ADHD resulting in a pooled prevalence of ADHD in CD of 1.4%. The meta-analysis of the two case-controlled studies is summarized in a forest plot in Figure 3a, the odds of having ADHD was significantly higher in the CD groups compared to controls (OR 1.39, 95% CI 1.18–1.63, *p* < 0.0001). Figure 3b shows a funnel plot for these studies. The prevalence of CD in ADHD was investigated by two cross sectional studies [45,46]. One out of 401 ADHD participants was diagnosed with CD, making the pooled prevalence of CD in ADHD 0.3% [45].

#### 3.1.1. GFD in ASD and ADHD

Two articles examined the role of the GFD in ASD, both observing no significant differences in behavioural symptoms between participants adhering to a GFD and those who did not [33,37]. Similarly, two articles examined the role of GFD in ADHD [40,41]. Both found significant improvements in behavioural symptoms, however, both studies are based on small sample sizes.

#### 3.1.2. Limitations of Studies in ADHD and ASD

Firstly, several studies had small sample sizes [35,36,37,38,39,40,41,47]. This is especially important due to the heterogeneity of ASD, and therefore there is a particular need for large sample sizes [31]. However not all of the studies suffered from this limitation, Butwicka et al. (2017) and Ludvigsson et al. (2013) consisted of very large sample sizes, and therefore results held statistical power.

Secondly, not all of these studies controlled for patients being already on GFD, Ludvigsson et al. (2013) emphasises the importance of this, as it can cause levels of gluten related antibodies to fall resulting in false negative CD diagnosis. This is particularly relevant to ASD, because of high numbers of individuals with ASD adhering to a GFD [31]. Thirdly, despite much deliberation surrounding increases in GI symptoms and intestinal permeability in ASD mentioned in these articles, only two tested these theories [38,39]. Lastly, it is also worth considering that the majority of these studies for both ASD and ADHD concern pediatric populations, which is understandable as ASD and ADHD are both prevalent in childhood [31,34,35,37,38,39,40,41,42,45,46]. However, unlike ASD and ADHD, CD is not confined to childhood and is very prevalent later on in life [45].

### 3.2. Mood Disorders

This group of disorders contained the largest number of articles eligible for inclusion. Twenty articles in total, all investigating mood disorders in CD patients, accounted for a total of 16,412 participants [34,41,43,44,47,48,49,50,51,52,53,54,55,56,57,58,59,60,61]. Ten studies suggested a significant association between mood disorders and CD [34,47,48,49,50,51,52,53,54,55]. The majority of these studies were concerned with depression, with the second most common being anxiety.

#### 3.2.1. Depression

**Figure 4:**
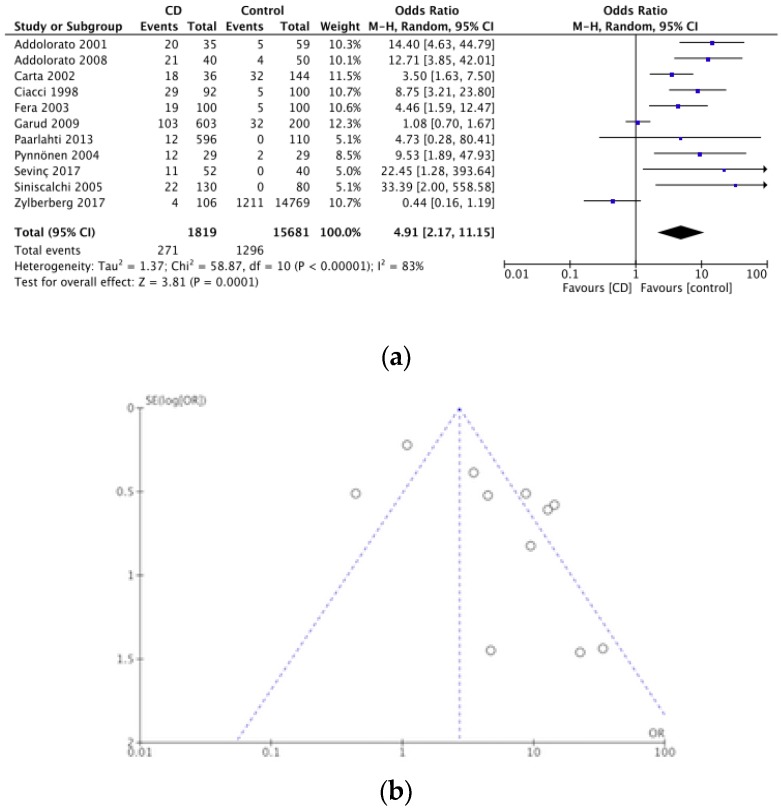
(**a**) Forest plot of pooled prevalence of depression in CD. (**b**) Funnel plot investigating distribution in depression studies.

Nineteen studies evaluated the presence of depression in CD patients. Out of a total of 16,300 participants, depression was found in 565. The prevalence of depression in CD was assessed by 11 case-controlled studies [44,51,52,53,54,55,56,57,58], 4 cross-sectional studies [43,47,59,60], 2 case series [52,61], and 2 cohort studies [49,50]. The pooled prevalence of depression in CD was 3.5%. The meta-analysis of the 11 case-controlled studies is summarized in a forest plot in Figure 4a, the odds of having depression was significantly higher in the CD groups compared to controls (OR 2.17, 95% CI 2.17–11.15, *p* < 0.0001). Figure 4b shows a funnel plot for these 11 studies.

#### 3.2.2. Anxiety

**Figure 5:**
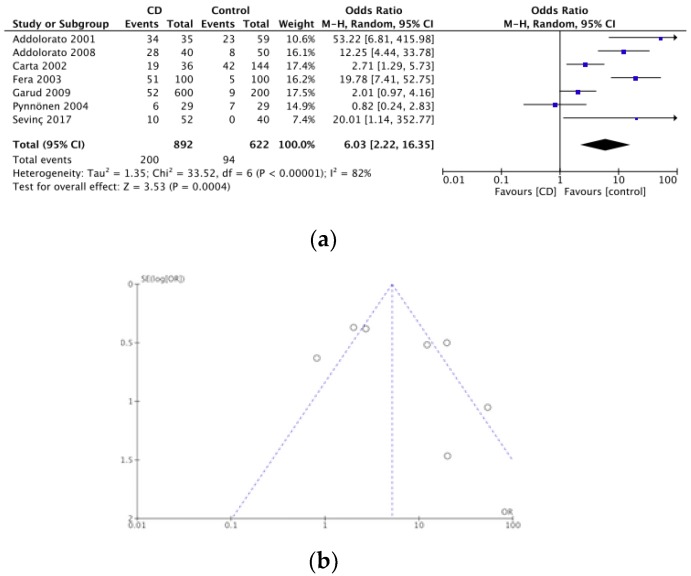
(**a**) Forest plot of pooled prevalence of anxiety in CD. (**b**) Funnel plot investigating distribution in anxiety studies.

Ten articles assessed anxiety in CD patients. Out of a total of 11,884 participants there were 443 cases of anxiety. The pooled prevalence was investigated by one cross sectional study [47] eight case-controlled studies [34,44,48,52,53,54,55,58] and one case series study [41]. The pooled prevalence of anxiety in CD was therefore 3.7%. A meta-analysis of seven of these case-controlled studies is summarized in a forest plot in Figure 5a, the odds of having anxiety was significantly higher in the CD groups compared to controls (OR 6.03, 95% CI 2.22–16.35, *p* < 0.0001). Figure 5b represents a funnel plot for these studies.

#### 3.2.3. Bipolar Disorder

**Figure 6:**
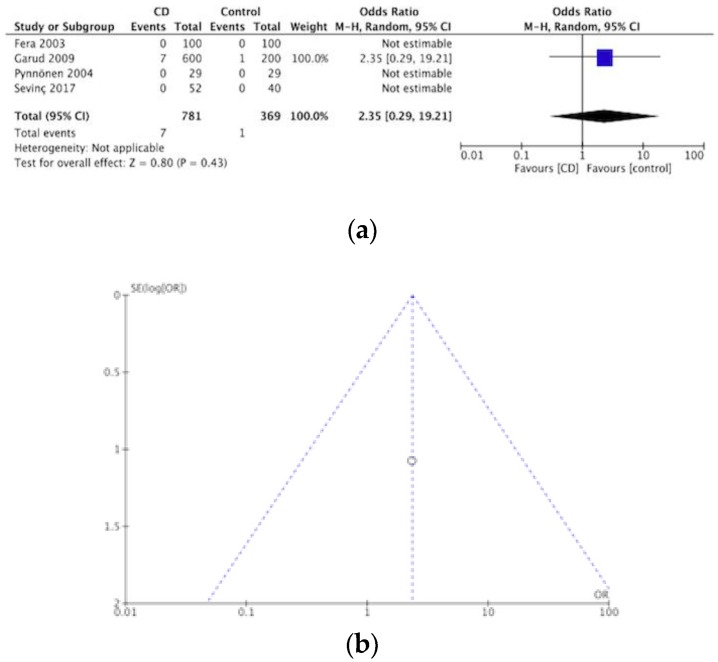
(**a**) Forest plot of pooled prevalence of bipolar disorder in CD. (**b**) Funnel plot investigating distribution in bipolar disorder studies.

Out of these articles, eight provided data concerning the prevalence of bipolar disorder in CD, this was made up of one cross sectional study [59] two case series studies [41,62] one cohort study [50], and four case-controlled studies [43,48,52,53]. These studies add up to 14,820 participants, 33 of these were found to already have, or meet the criteria, for a bipolar disorder diagnosis. Producing a pooled prevalence of bipolar disorder in CD of 0.2%. The meta-analysis of the four case-controlled studies is summarized in a forest plot in Figure 6a, no statistically significant differences were detected for bipolar disorder and CD, compared to controls (OR 2.35, 95% CI 2.29–19.21, *p* = 0.43). Figure 6b presents a funnel plot for these studies.

#### 3.2.4. GFD in Mood Disorders

Nine studies provided data concerning the role of the GFD, four of which reported no association between anxiety and depression with adherence to a GFD [48,53,56,58]. Some claimed that adhering to a GFD causes worsening or persistence of depressive symptoms [51,55,57]. Furthermore, Addolorato (2001) reported improved anxiety but sustained depression symptoms in patients on a GFD.

#### 3.2.5. Limitations in Studies of Mood Disorders

Firstly, self-reported measures of both anxiety and depression, as well as GFD adherence limit validity [59,60]. Additionally, much conflict exists in literature surrounding the duration of GFD. It has been argued that any effect of GFD should be investigated longitudinally after at least one year of a GFD, as otherwise results lack reliability [41,55]. This has not been the case for the majority of these studies. Also, several potential confounding factors are highlighted, that are not always controlled for, such as autoimmune thyroiditis, family history of mental illness, and severity of CD symptoms [52,60]. Additionally, cultural differences regarding the social burden of the GFD should also be taken into consideration. For example, in Italy this is likely to be more prominent, as food holds more cultural and social importance when compared to other countries [55,58].

### 3.3. Schizophrenia and Other Psychotic Disorders

**Figure 7:**
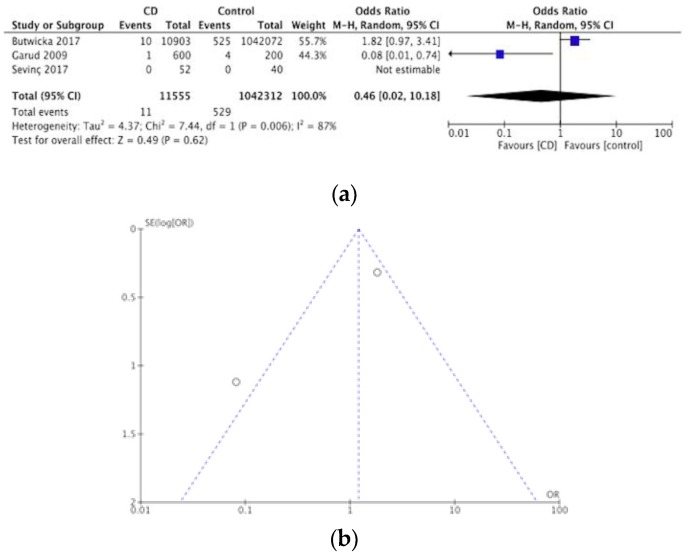
(**a**) Forest plot of pooled prevalence of schizophrenia and other psychotic disorders in CD. (**b**) Funnel plot investigating distribution in schizophrenia and other psychotic disorders studies.

A total of six articles investigated the prevalence of schizophrenia and other psychotic disorders in CD, adding up to a total of 11,741 participants. This assessment of the prevalence of schizophrenia and other psychotic disorders in CD was performed by one cross sectional study [43] four case-controlled studies [34,44,48,52] and one case series study [41]. Out of these a total of 12 were identified with either schizophrenia or another psychotic disorder, producing a pooled prevalence of schizophrenia and other psychotic disorders in CD of 0.1%. Ten of these came from Butwicka et al. (2017) who had a sample of 10,903 with CD, and then one each from Garud et al. (2009) and Vaknin et al. (2004). None of these studies specifically concluded that there was a significant association between schizophrenia or other psychotic disorders and CD. The meta-analysis of three of the case-controlled studies is summarized in a forest plot in Figure 7a, no statistically significant differences were detected with schizophrenia or other psychotic disorders and CD compared to controls (OR 0.46, 95% CI 0.02–10.18, *p* = 0.62). Figure 7b) displays a funnel plot for these studies.

#### 3.3.1. GFD in Schizophrenia and Other Psychotic Disorders

None of the studies examined the impact of the GFD in cases of schizophrenia and other psychotic disorders [41,48]. Therefore, there is very limited information on the subject.

#### 3.3.2. Limitations in Studies on Schizophrenia and Other Psychotic Disorders

This was the category with the least number of eligible articles, resulting in restricted information to interpret, which is a criticism in itself. Another limitation is that several studies were investigating cases of schizophrenia or other psychotic disorders in CD patients from pediatric populations [34,41,48,52]. This may be a significant limitation, as it is currently understood that the onset of schizophrenia or other psychotic disorders during childhood is uncommon and is instead most likely to present between adolescence and early adulthood.

### 3.4. Eating Disorders

**Figure 8:**
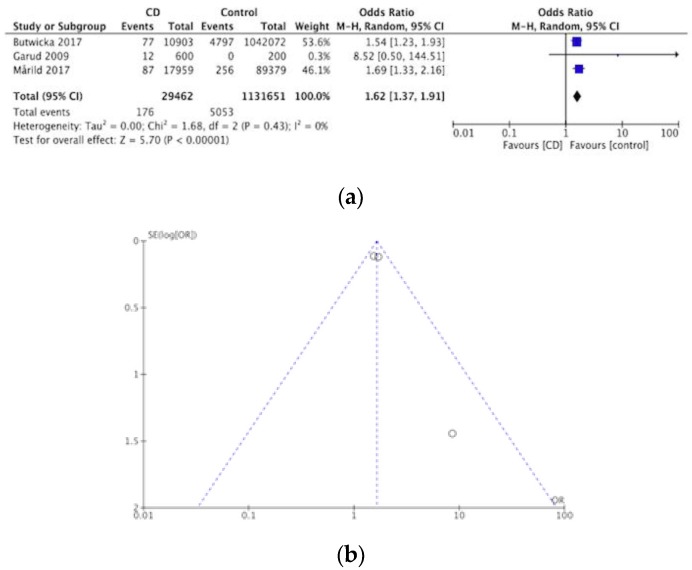
(**a**) Forest plot of pooled prevalence of eating disorders in CD. (**b**) Funnel plot investigating distribution in eating disorder studies.

Out of nine articles concerning eating disorders and CD, four concluded that there is a significant association. The prevalence of eating disorders within CD was investigated by one cohort and case control study [63] four case control studies [34,44,48,52] and three cross sectional studies [43,64,65]. Out of 29,977 CD patients, coexisting eating disorders were detected in 221, creating a pooled prevalence of eating disorders in CD of 0.7%. The meta-analysis of the three case-controlled studies is summarized in a forest plot in Figure 8a, the odds of having an eating disorder was significantly higher in the CD groups compared to controls (OR 1.62, 95% CI 1.37–1.91, *p* < 0.00001). Figure 8b shows a funnel plot for these studies.

The prevalence of CD within eating disorders was assessed by one cross sectional study [65] and one case-controlled study [66,67]. Amongst 841 patients with eating disorders, 15 cases of CD were determined, therefore the pooled prevalence of CD in eating disorders was 1.8%.

#### 3.4.1. GFD in Eating Disorders

Only one study that explored associations between eating disorders and CD also investigated the impact of the GFD [66]. This study obtained a sample where all participants had a diagnosis of anorexia nervosa, one participant was found to also have CD. No differences in disordered eating were found whilst following a GFD, however resolution of amenorrhea was noted in this individual [66].

#### 3.4.2. Limitations in Studies on Eating Disorders

Only two out of these nine articles had a sufficiently large sample size [34,63]. As a result, the reliability of results in studies with smaller samples risk being compromised. Another limiting factor is gender, as male participants were often excluded [63,64,67]. Even in those featuring male participants, male sample sizes were always very small. [64,66,67]. Welch, Ghaderi, and Swenne (2015) acknowledge this in their study. Lastly, screening for CD requires ingestion of sufficient amounts of gluten in order to avoid false negative results. This risk is much higher in participants with eating disorders [66].

## 4. Discussion

This systematic review has identified a significant increased risk for ASD, ADHD, depression, anxiety, and eating disorders amongst patients with CD compared to healthy controls. No significant risk was identified for bipolar disorder or schizophrenia.

Clearly such findings are relevant to clinical practice, as both ASD and ADHD patients are often advised to adopt a GFD to reduce behavioural problems [38,40,41,42,43,44,45,46]. There is no rational for doing so unless the patient has been tested for CD prior to adopting a GFD. There is an urgent need for studies investigating the effects of a GFD in these populations as what has been published so far has not been adequately powered, the duration of the intervention was suboptimal and the monitoring of the strictness of adherence to a GFD using repeat serological testing was not undertaken [33,37].

Associations between CD and neurodevelopmental disorders could suggest an unknown biological cause with some invoking the gut–brain axis relationship [34,37]. However, such biological explanations lack evidence, therefore further research is required [33,39]. Of interest is the role of the cerebellum in ASD and ADHD. The cerebellum has emerged as one of the key brain regions affected in non-motor disorders, including autism spectrum disorder and attention deficit-hyperactivity disorder. The cerebellum is the principle brain target in both CD and gluten sensitivity.

Examining the prevalence of depression and anxiety demonstrated significant increased risk in CD patients compared to controls. This is in keeping with anecdotal reports from health professionals that care for patients with CD, that both anxiety and depression are prominent features in this group. No statistically significant differences were identified for bipolar disorder in CD patients. Research often distinguishes between pre and post CD diagnosis to draw hypotheses concerning anxiety and depression in CD, claiming adherence to a GFD causes anxiety to subside whilst depression often persists [34,55]. Social implications of the GFD (social isolation, avoiding going out because of the risk of contamination, having to always declare the condition amongst friends and colleagues, having to explain the diagnosis of CD as opposed to a life choice of GFD, etc.) are blamed for this [34,47,50,51,55,56,59]. Psychological support beyond simply advising a GFD is argued in several studies, may promote acceptance and subsequent adherence to the GFD, as well as reducing the risk of anxiety and depression [47,53,57].

Our meta-analysis assessing the prevalence of schizophrenia and other psychotic disorders in CD patients, found no significant difference compared to healthy controls. However, a portion of the wider literature still argues for an association [68,69]. There are several case reports reporting patients with acute psychosis developing at the same time as a diagnosis of CD being made. The argument in favour of a link is based on the fact that these patients seem to improve on a strict GFD. The identification of immune mediated psychosis in the context of NMDA encephalitis for example, also provides some evidence for autoimmunity having a role in these disorders.

Significantly increased prevalence of eating disorders in CD patients was detected in this meta-analysis. Theories often relate back to the vigilance required for a GFD, as this may produce a fixation with food intake as a whole [63,64]. The prominent gastrointestinal symptoms that can be seen in the context of CD may also play a part in driving the fear of eating. The risk of misdiagnoses due to similarities between eating disorders and CD symptoms is often discussed in literature, for example GI and malnutrition symptoms are present in both [63,66]. For this reason, monitoring and awareness of the possibility of CD is described as crucial [63,64,66,67].

Associations between depression, anxiety, and eating disorders are apparent as a result of the psychological and social implications of CD, however specific biological causes for these disorders are uncertain. Psychological and social implications are less clear for ASD, ADHD, where biological causes are speculated to play a more prominent role. Further research is required to add clarity to what seems to be a rather conflicting literature.

## 5. Limitations

There was a significant heterogeneity between studies included in this review which is reflected in the funnel plots. This could be explained by the existence of grey literature or simply might reflect the fact that the subject is still understudied and that more studies should be carried out in the future.

Secondly, a single database was utilized to conduct the literature search for this study. This may have caused some studies to be excluded. However, we have checked the reference lists of every included study to identify additional seminal publications.

Finally, the role of GFD has been studied but only in observational studies, the majority of which were conducted in small populations. By definition, observational studies provide low evidence and therefore no recommendations can be made based on this review [70]. However, RCTs on the matter might shed further light into the matter.

## 6. Conclusions

The findings for this systematic review and meta-analysis provide support for the notion that CD has an increased risk for specific psychiatric disorders probably through indirect adverse effects on mental health and social life. However further research is required to investigate the pathophysiology of such associations.

## Figures and Tables

**Table 1:** Characteristics of studies included in this review

Parameter	Value
Number of papers	37
Population (%)	
Adult	32
Children	46
Mixed	22
Type of study	
Cohort	2
Case-controlled	18
Cohort and Case-controlled	1
Cross-sectional	14
Psychiatric disorder	
ASD	9
ADHD	8
Mood disorders	20
Schizophrenia and other psychotic disorders	6
Eating disorders	9
Year of publication (%)	
Until 2000	5
2000–2009	43
2010–2019	51
